# The impact of aspirin use on outcomes in patients with inflammatory bowel disease: Insights from a national database

**DOI:** 10.1007/s00384-023-04575-7

**Published:** 2023-12-20

**Authors:** Humzah Iqbal, Gagandeep Singh Arora, Ishandeep Singh, Isha Kohli, Hunza Chaudhry, Aalam Sohal, Devang Prajapati

**Affiliations:** 1 Department of Internal Medicine, University of California San Francisco, Fresno, USA; 2 Department of Internal Medicine, University of California Riverside, Riverside, USA; 3 Dayanand Medical College and Hospital, Ludhiana, India; 4 Icahn School of Medicine at Mount Sinai, New York City, USA; 5 Liver Institute Northwest, Seattle, USA; 6 Department of Gastroenterology and Hepatology, University of California San Francisco, Fresno, USA

**Keywords:** Aspirin, Inflammatory bowel disease, Crohn’s disease, Ulcerative colitis, Nationwide inpatient sample.

## Abstract

**Background:**

Inflammatory bowel disease (IBD) is an inflammatory disorder that can increase the risk of mortality. Aspirin is an anti-inflammatory drug used for primary prevention of cardiovascular events. A single center analysis previously reported that aspirin use did not impact major outcomes in IBD. In this study, we aim to assess the impact of aspirin use on mortality and other outcomes in patients with IBD using national data.

**Methods:**

National inpatient sample (NIS) 2016–2020 was used to identify adult patients with IBD. Data were collected on patient demographics, hospital characteristics, and comorbidities. The outcomes studied were in-hospital mortality, sepsis, shock, Intensive Care Unit (ICU) admission, and need for surgery. Multivariate logistic regression analysis was performed.

**Results:**

A total of 1,524,820 IBD hospitalizations were included. Of these, 137,430 (9%) were long-term aspirin users. The majority of the patients in the aspirin group were aged > 65 years (34.11%), female (56.37%), White (78.83%) and had Medicare insurance (36.77%). Aspirin users had a lower incidence of in-hospital mortality (1.6% vs 1.4%, P = 0.06), sepsis (2.5% vs 2.9%, P < 0.001), shock (2.9% vs 3.4%, P < 0.001), ICU admission (2.6% vs 2.9%, P < 0.001), need for surgery (2.1% vs 4.2%, P < 0.001). After adjusting for confounders, aspirin was associated with a reduction in mortality (adjusted odds ratio: 0.49, 95%CI 0.45–0.55, P < 0.001).

**Conclusion:**

Our study reports that aspirin use among patients with IBD was associated with a lower risk of death, sepsis, and shock. Aspirin use may have a protective effect in patients with IBD. Further studies are needed to confirm these results.

**Supplementary Information:**

The online version contains supplementary material available at 10.1007/s00384-023-04575-7.

## Introduction

Inflammatory bowel disease (IBD) is a chronic inflammatory disorder of the gastrointestinal tract that consists of Crohn’s disease (CD) and ulcerative colitis (UC). IBD is a significant cause of morbidity, affecting over 1 million people in the United States (US) [1]. IBD is also associated with an increased rate of short-term and long-term mortality [2]. CD and UC are both complex disease states with microbial, genetic, immune, and environmental factors [3]. The relationship between IBD and cardiovascular disease is currently under debate, however, previous literature has shown that the systemic inflammation induced by IBD may put patients at an increased risk of developing atherosclerosis, ischemic heart disease, and thromboembolism [4].

Due to the increased risk of atherosclerosis, it is likely that the aspirin use will continue to increase in this population. Aspirin is a cyclooxygenase (COX) 1 and COX 2 inhibitor that is well-validated and exceedingly common in the prevention of cardiovascular events [5]. Over 60 million people engage in regular non-steroidal anti-inflammatory drug (NSAID) use in the US, and many of them take daily low dose aspirin [6]. Aspirin has been associated with mixed results regarding gastrointestinal (GI) pathology, with some studies demonstrating an increased risk for mucosal irritation and GI bleed, particularly in older patients [7, 8]. However, a meta-analysis by Bosetti et al. found that long-term aspirin use is associated with a decreased risk of several GI malignancies, including colorectal cancer [9]. NSAIDs including aspirin have shown conflicting results in patients with IBD, with some studies suggesting that they may be associated with an increased risk of disease flare, while others have found no effect [10].

A single-center retrospective analysis of 764 IBD patients by Patel et al. found that there was no difference in major clinical outcomes including IBD-related surgery among daily aspirin users versus non-aspirin users [11]. Till now, no study has assessed the impact of long-term aspirin use on outcomes in patients with IBD. In this study, we sought to assess the effects of long-term aspirin use on outcomes of IBD using a large national database.

## Methods

### Data source

The National Inpatient Sample (NIS) database, administered by the Agency for Healthcare Research and Quality (AHRQ), is the largest inpatient database in the US. It contains data from 20% of all hospitalizations in the US, representing approximately 8 million (unweighted) and 40 million (weighted) hospitalizations yearly. It contains one primary diagnosis, up to 40 secondary diagnoses, population baseline characteristics, patient comorbidities, and total charges [12].

### Ethical statements

We conducted this study in compliance with the principles of the Declaration of Helsinki. Due to the nature of the NIS database, all patient data is completely de-identified. Therefore, institutional review board (IRB) approval and written consent was not required.

### Study population

**Fig. 1:**
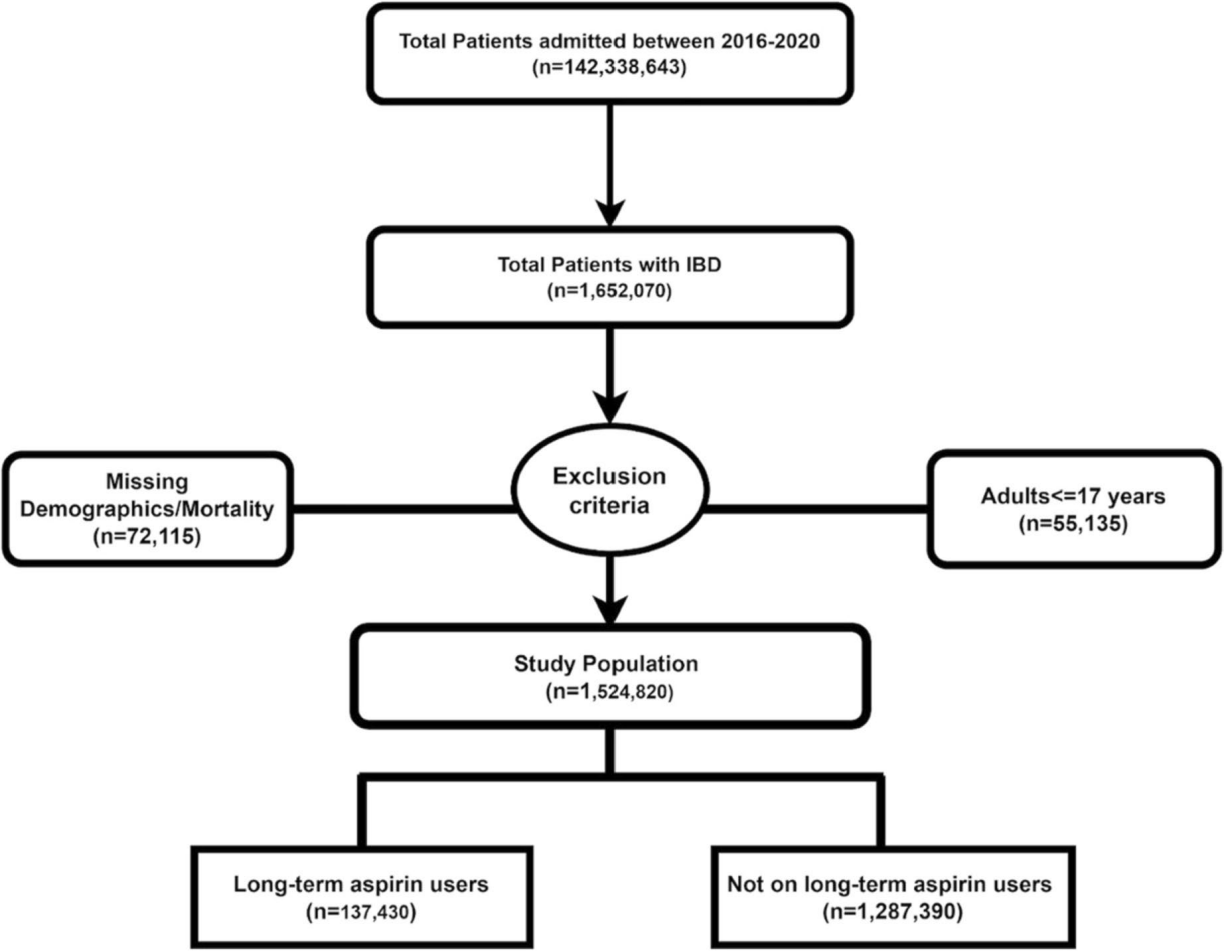
Flow diagram of inclusion process

The NIS database from 2016 to 2020 was queried according to the *International Classification of Diseases, Tenth Revision* (*ICD-10*), and *Clinical Modification* for patients with a primary or secondary hospitalization diagnosis of IBD. ICD10-code K50 was used for CD and K51 for UC. Patients were divided into two groups based on the presence of aspirin. ICD-10 code Z79.82 was used to query patients with a concomitant diagnosis of long-term (current) aspirin use. This method has been used in previous studies to identify patients with long-term aspirin use [13–18]. Patients who were under 18 or missing information on demographics/mortality were excluded from the analysis. A total of 1,524,820 hospitalizations were included in the analysis. This is depicted in Fig. 1. We further performed sub-group analysis by including only patients with IBD without any cancer; only UC; only CD; complicated IBD; and IBD with colorectal cancer.

### Study variables

Data were collected on patient demographics (age, sex, race, primary insurance, and income quartile), hospital characteristics (location, region, teaching status, and bed size of the hospital), and the Charlson Comorbidity index (CCI). Information was also collected regarding the complications of IBD. Complicated IBD was defined as either CD or UC with the presence of fistula (ICD-10 K50.013; K50.113; K50.813; K50.913; K51.013; K51.213; K51.313; K51.913), abscess (ICD-10 K50.014; K50.114; K50.814; K50.914; K51.014; K51.214; K51.314; K51.914, or bleed (ICD-10 K50.011; K50.111; K50.811; K50.911; K51.011; K51.211; K51.311; K51.911).

### Outcomes

The primary study outcome was in-hospital mortality. Secondary study outcomes were sepsis, shock, acute kidney injury (AKI), intensive care unit (ICU) admission, blood transfusion (BT) requirement, and abdominal surgery. Information was also collected on surrogate markers of resource utilization, such as length of stay (LOS) in days, and total hospitalization charges (THC). Hospital charges are defined as the dollar amount the hospital charges for services before negotiating discounts with insurance companies.

### Statistical analysis

Data is presented as population-weighted mean ± SE for continuous variables and the total number of patients with percentages for categorical factors. The variables included in the univariate analysis were patient characteristics, hospital characteristics, complicated IBD and Charlson comorbidities. CCI is a validated scoring system used to assess and standardize comorbidity burden and resource utilization based on disease severity [19]. Only variables noted to have a p < 0.1 were included in the multivariate regression model. Multivariate logistic regression analysis was then performed to assess the impact of long-term aspirin on outcomes in patients with AP. A p-value < 0.05 was considered to be statistically significant. The results were expressed in adjusted odds ratio (aOR) with a 95% confidence interval (CI).

## Results

### Patient demographics and hospital characteristics

A total of 1,524,820 hospitalizations were included in the study. Of these, 137,430 (9.01%) patients were aspirin users. The majority of the patients in the aspirin group were elderly > 65 years of age (64.8%), female (51%), had Medicare (68.8%), and were in the second income quartile (26.5%). A complete list of demographic differences between taking aspirin and those not on aspirin is presented in Table 1.

**Table 1:** Baseline patient characteristics, stratified by long-term aspirin use

	**Absence of Aspirin n (%)**	**Presence of Aspirin n (%)**	**p- value**
**Age category**			** < 0.001**
18–44	510,765 (36.8)	7,315 (5.3)	
45–64	445,510 (32.1)	41,070 (29.9)	
> 65	431,115 (31.1)	89,045 (64.8)	
**Sex**			** < 0.001**
Males	597,685 (43.1)	67,475 (49.1)	
Females	789,705 (56.9)	69,955 (51)	
**Race**			** < 0.001**
White	1,084,745 (78.2)	117,285 (85.3)	
Black	159,715 (11.5)	11,480 (8.3)	
Hispanic	88,310 (6.4)	4,805 (3.5)	
Asian/Pacific Islander	16,990 (1.2)	1,400 (1)	
Native American	5,260 (0.4)	415 (0.3)	
Other	32,370 (2.3)	2,045 (1.5)	
**Primary expected payer**			** < 0.001**
Medicare	560,770 (40.4)	94,550 (68.8)	
Medicaid	215,520 (15.5)	8,980 (6.5)	
Private	517,530 (37.3)	29,120 (21.2)	
Uninsured	52,015 (3.7)	1,735 (1.3)	
**Median household income**			**0.002**
Lowest quartile	342,280 (24.7)	32,970 (24)	
Second quartile	353,670 (25.5)	36,365 (26.5)	
Third quartile	356,765 (25.7)	35,820 (26.1)	
Highest quartile	334,675 (24.1)	32,275 (23.5)	
**Region of hospital**			** < 0.001**
Northeast	307,810 (22.2)	27,205 (19.8)	
Midwest	327,420 (23.6)	40,025 (29.1)	
South	517,475 (37.3)	48,720 (35.4)	
West	234,685 (16.9)	21,480 (15.6)	
**Hospital Location**			** < 0.001**
Rural	96,305 (6.9)	11,100 (8.1)	
Urban	1,291,085 (93.1)	126,330 (91.9)	
**Teaching status of the hospitals**			** < 0.001**
Non-teaching Hospitals	365,290 (26.3)	38,325 (27.9)	
Teaching Hospitals	1,022,100 (73.7)	99,105 (72.1)	
**Bed size of hospital**			** < 0.001**
Small	276,335 (19.9)	29,060 (21.1)	
Medium	386,895 (27.9)	38,745 (28.2)	
Large	724,160 (52.2)	69,625 (50.7)	
**CCI**			** < 0.001**
0	639,910 (46.1)	24,455 (17.8)	
1	282,760 (20.4)	30,640 (22.3)	
2	172,975 (12.5)	27,150 (19.8)	
≥ 3	291,745 (21)	55,185 (40.1)	
**Complicated IBD**	307,265 (22.2)	19,355 (14.1)	** < 0.001**

Bold print signifies statistical significance

### Underlying comorbidities

Patients in the aspirin group had a higher prevalence of acute myocardial infarction, congestive heart failure, peripheral vascular disorders, cerebrovascular disease, dementia, chronic obstructive pulmonary disease (COPD), rheumatoid disease, peptic ulcer disease, diabetes, diabetes with complications, hemiplegia/paraplegia, Acquired immunodeficiency syndrome/ human immunodeficiency virus (AIDS/HIV), renal disease and cancer compared to patients who were not on aspirin. A complete list of underlying comorbidities between the two groups is presented in Supplementary Table 1.

### Outcomes

The incidence of in-hospital mortality among aspirin users was 2,060 (1.5%), while in the non-aspirin group it was noted to be 22,925 (1.6%). The complete list of in-hospital outcomes stratified by aspirin use is presented in Table 2. After adjusting for confounding factors aspirin use was associated with 51% lower odds of in-hospital mortality compared to non-aspirin group (aOR 0.49, 95%CI 0.45–0.55, P < 0.001). The complete results of multivariate regression analysis after adjusting for confounders is presented in Table 3. The incidence of sepsis among aspirin users was 3,430 (2.5%), while in the non-aspirin group was noted to be 41,330 (2.9%). After adjusting for confounding factors aspirin use was associated with 28% lower odds of sepsis compared to non-aspirin group (aOR 0.72, 95%CI 0.67–0.78, P < 0.001). The incidence of shock among aspirin users was 3,950 (2.9%), while in the non-aspirin group was noted to be 47,135 (3.4%). After adjusting for confounding factors aspirin use was associated with 47% lower odds of shock compared to non-aspirin group (aOR0.53, 95%CI 0.49–0.58, P < 0.001). The incidence of AKI among aspirin users was 28,690 (20.1%), while in the non-aspirin group was noted to be 224,800 (16.2%). After adjusting for confounding factors aspirin use was associated with 23% higher odds of AKI compared to non-aspirin group (aOR 1.23, 95%CI 1.20–1.26, P < 0.001). The incidence of ICU admissions among aspirin users was 3,650 (2.6%), while in the non-aspirin group was noted to be 41,185 (2.9%). After adjusting for confounding factors aspirin use was associated with 46% lower odds of ICU admissions compared to non-aspirin group (aOR 0.54, 95%CI 0.50–0.59, P < 0.001). The incidence of blood transfusions among aspirin users was 445 (0.3%), while in the non-aspirin group was noted to be 5,460 (0.4%). After adjusting for confounding factors aspirin use was associated with 31% lower odds of blood transfusions compared to non-aspirin group (aOR 0.69, 95%CI 0.54–0.87, P = 0.002). The incidence of abdominal surgeries among aspirin users was 2,940 (2.1%), while in the non-aspirin group was noted to be 58,225 (4.2%). After adjusting for confounding factors aspirin use was associated with 23% lower odds of abdominal surgeries compared to non-aspirin group (aOR 0.77, 95%CI 0.70–0.84, P < 0.001).

**Table 2:** In-hospital outcomes, stratified by aspirin use

**Outcomes**	**Absence of Aspirin n (%)**	**Presence of Aspirin n (%)**	**p- value**
Mortality	22,925 (1.6)	2,060 (1.5)	0.06
Sepsis	41,330 (2.9)	3,430 (2.5)	** < 0.001**
Shock	47,135 (3.4)	3,950 (2.9)	** < 0.001**
AKI	224,800 (16.2)	28,690 (20.1)	** < 0.001**
ICU	41,185 (2.9)	3,650 (2.6)	**0.005**
BT	5,460 (0.4)	445 (0.3)	0.08
Surgery	58,225 (4.2)	2,940 (2.1)	** < 0.001**
Length of Stay	5.4(+/-0.2)	4.86(+/-0.4)	** < 0.001**
Total hospitalization charges	$58,523.2 (+/-468)	$56,404.2 (+/-581.7)	** < 0.001**

Bold print signifies statistical significance

**Table 3:** Results of multivariate logistic regression analysis, after adjusting for confounding factors

Outcome	Odds Ratio	95% Confidence Interval	p-value
Death	0.49	0.45–0.55	** < 0.001**
Sepsis	0.72	0.67–0.78	** < 0.001**
Shock	0.53	0.49–0.58	** < 0.001**
AKI	1.23	1.20–1.26	** < 0.001**
ICU	0.54	0.50–0.59	** < 0.001**
BT	0.69	0.54–0.87	**0.002**
Surgery	0.77	0.70–0.84	** < 0.001**
Length of Stay	-1.06	-1.14- -1	** < 0.001**
Total hospitalization charges	-10950.31	-12076.7- -9823.9	** < 0.001**

Variables listed as covariates in final multivariate logistic regression included age, sex, race, insurance status, income quartile, comorbidities, complicated IBD, and hospital characteristics

Bold print signifies statistical significance

The length of stay among aspirin users was 4.86 (+/-0.4), while in the non-aspirin group was noted to be 5.4(+/-0.2). After adjusting for confounding factors aspirin use was associated with lower odds of length of stay compared to non-aspirin group (adjusted coefficient -1.06, 95%CI -1.14- -1, P < 0.001). The total hospitalization charges among aspirin users was $56,404.2 (+/-581.7), while in the non-aspirin group was noted to be $58,523.2 (+/-468). After adjusting for confounding factors aspirin use was associated with lower odds of total hospitalization charges compared to non-aspirin group (adjusted coefficient -10950.31, 95%CI -12,076.7- -9823.9, P < 0.001).

### Sub-group analysis

#### Patients with IBD without cancer

A total of 1,422,350 patients with IBD did not have cancer. There were 127,945 patients in the aspirin group. After adjusting for confounding factors, patients on long-term aspirin use had a statistically significant lower odds of mortality (aOR 0.50, 95% CI 0.45–0.56, P < 0.001). Patients in the aspirin group had statistically significant lower length of stay (adj. Coeff -1.03 days, 95% CI -1.11 - -0.96, P < 0.001) and total hospitalization charges (adj. Coeff -$10247, 95% CI -11402.7 - -9092, P < 0.001).

#### Patients with UC

A total of 582,030 patients with UC were included in the analysis. There were 60,145 patients in the aspirin group. Similar results were noted in this subgroup with long-term aspirin use being associated with lower odds of in-hospital mortality (aOR 0.47, 95% CI 0.40–0.54, P < 0.001), shorter length of stay (adj. Coeff -1.22 days, 95% CI -1.33-1.12, P < 0.001) and lower total hospitalization charges (adj. Coeff -$13,583, 95% CI -15,326.2 - -11,839, P < 0.001).

#### Patients with CD

A total of 942,790 patients with CD were included in the analysis. There were 77,285 patients in the aspirin group. After adjusting for confounding factors, long-term aspirin use was associated with lower odds of in-hospital mortality (aOR 0.52, 95% CI 0.48–0.57, P < 0.001), shorter length of stay (adj. Coeff -0.95 days, 95% CI -1.04 - -0.85, P < 0.001) and lower total hospitalization charges ( adj. Coeff -$8,965, 95% CI -10,359 - -7,571, P < 0.001).

#### Patients with complicated IBD

A total of 326,620 patients had complicated IBD. There were 19,355 patients in the aspirin group. After adjusting for confounding factors, patients in the long-term aspirin use group had lower odds of in-hospital mortality (aOR 0.44, 95% CI 0.32–0.59, P < 0.001), shorter length of stay (adj. Coeff -1.35 days, 95% CI -1.57 - -1.12, P < 0.001) and lower total hospitalization charges ( adj. Coeff -$17,224, 95% CI -20,414 - -14,033, P < 0.001).

#### Patients with IBD and colorectal cancer

A total of 9,505 patients had IBD and colorectal cancer. There were 665 patients on long-term aspirin. Long-term aspirin use was not associated with in-hospital mortality (aOR 0.80, 95% CI 0.30–2.13, P = 0.66), but had shorter length of stay (adj. Coeff -1.42 days, 95% CI -2.49 - -0.35, P = 0.009) and lower total hospitalization charges (adj. Coeff -$17,224, 95% CI -20,414 - -14,033, P < 0.001).

## Discussion

Our study used a large national inpatient database to assess the outcomes of patients hospitalized with IBD in patients taking aspirin. IBD has been linked to increased risk for developing cardiovascular disease due to systemic inflammation [4]. Our study noted that approximately 9% of hospitalized patients with IBD were on long-term aspirin. Majority of patients were older in age, females and White which is consistent with the patient population most likely to be taking long-term aspirin [20–22]. Previous literature has shown that White patients are more likely to be taking long-term aspirin than Black and Hispanic patients, even when controlling for cardiovascular risk factors [22]. These findings are helpful as they suggest that our study sample is representative of the population taking aspirin.

Our study noted that in-hospital mortality among aspirin users was lower than patients not taking aspirin. Previous studies have shown mixed results regarding aspirin use in patients with IBD. Some studies have shown no difference in major clinical outcomes including death among aspirin users [11]. A single-center retrospective analysis of 764 IBD patients by Patel et al. found that there was no difference in major clinical outcomes including IBD-related surgery among daily aspirin users versus non-aspirin users [11]. Our study is the first to report mortality benefit among hospitalized patients with IBD. We also found a similar mortality benefit on our sub-group analysis separately in UC patients, CD patients, patients with complicated IBD, and patients with IBD while excluding those with cancer.

Our study found higher odds of AKI among patients with long term aspirin use. Both traditional and selective NSAIDs have been shown to have an association with AKI [23]. A case–control study by Lafrance et al. found an increased risk of AKI development with less selective NSAIDs such as aspirin and naproxen compared to more selective NSAIDs. Our study is consistent with previous literature, showing increased odds of AKI in IBD patients who are taking aspirin [24].

Our study also found lower odds of sepsis and shock, among patients taking long term aspirin compared to those without. These results are conflicting with the already available literature as aspirin use has been shown to damage GI mucosa and lead to earlier and more severe relapse in IBD [3, 25]. Other studies have reported that aspirin use may confer decreased disease activity in IBD, although the mechanism of this continues to be unclear. Interestingly, studies have shown that aspirin use decreases the risk of colorectal cancer (CRC) in IBD patients [26–29]. Further studies are needed to determine the exact mechanism of the protective effects of aspirin in IBD. IBD patients have been shown to have a higher risk of cardiovascular events, however, the anti-platelet effects of aspirin may mitigate this risk which can potentially contribute to the mortality benefit found in our study [30].

Our study also found lower odds of surgical intervention in patients with long term aspirin use. This finding may be due to the effect of aspirin on the disease activity among IBD patients. This hypothesis is further strengthened by the finding of lower rates of complicated IBD and lower need for blood transfusion in aspirin users compared to non-aspirin users. It is pertinent to note that in our study, the incidence of surgical intervention was 4%. Previous studies have shown that 20–30% of patients with UC and 30–40% of patients with CD will require surgery at some point in their disease course [31]. Our study finding of lower incidence is due to the nature of the database limiting our ability to identify surgeries that occurred after discharge, or later in the disease course.

Our study noted that aspirin users had lower length of stay and resource utilization compared to non-aspirin users, including in all sub-groups of our sub-group analysis. This could be due to the lower disease activity noted in aspirin users, which translated into lower need for ICU admission, surgery and blood transfusion. It has been reported previously that the use of invasive interventions is associated with increased healthcare cost and our findings overall suggests benefit of using aspirin in patients with IBD.

We acknowledge the following limitations of our study. Hospital readmissions cannot be tracked due to the nature of the NIS database. We are unable to track the patient after the hospitalization and as a result we cannot follow the patient longitudinally and therefore, cannot identify readmissions or surgical interventions performed later during the course. The cause of death is not identifiable, making the ultimate reason for mortality unclear. Additionally, NIS relies on proper documentation of ICD-10 codes, which are subject to human error. Patients on long-term aspirin were identified on the basis of ICD-10 coding. It is unclear whether this code captures all patients chronically taking aspirin, however several previous studies have also used this ICD-10 code as a valid measure to assess for aspirin usage [13–18]. Because of the nature of the database, we cannot identify for how long the patient was on aspirin, which may confound analysis. We are also unable to determine whether patients were on aspirin for primary versus secondary prophylaxis. The strength of the study includes large sample size and exclusion of regional bias. Despite the limitations, our study’s primary finding that aspirin use might have beneficial effects and may alter the disease course of IBD warrants attention.

In conclusion, our study found that aspirin use may be associated with improved outcomes in hospitalized patients with IBD. Further studies aiming to identify underlying mechanisms responsible for these findings will be of interest.

## Supplementary Information

Below is the link to the electronic supplementary material.

Supplementary file1 (DOCX 13 KB)

## Data Availability

All data for this study is available within the manuscript.
