# Supplementary material for: The impact of aspirin use on outcomes in patients with inflammatory bowel disease: Insights from a national database
Source: Int J Colorectal Dis. 2023 Dec 20;39(1):6. doi: 10.1007/s00384-023-04575-7 (PMC10733222; doi:10.1007/s00384-023-04575-7)
Supplement: Supplementary file 1 — Supplementary file1 (DOCX 13 KB) [file 384_2023_4575_MOESM1_ESM.docx]

Supplementary table 1. Patient comorbidities, stratified by long-term aspirin use

| **Underlying comorbidity** | **Absence of Aspirin n (%)** | **Presence of Aspirin n (%)** | **p- value** |
| --- | --- | --- | --- |
| History of acute myocardial infarction | 63,725 (4.6) | 25,275 (18.4) | **<0.001** |
| Congestive heart failure | 145,155 (10.5) | 33,315 (24.2) | **<0.001** |
| Peripheral Vascular Disorders | 71,510 (5.1) | 19,320 (14.1) | **<0.001** |
| Cerebrovascular disease | 44,320 (3.2) | 12,445 (9) | **<0.001** |
| Dementia | 42,100 (3) | 8,790 (6.4) | **<0.001** |
| COPD | 286,920 (20.7) | 40,925 (29.8) | **<0.001** |
| Rheumatoid disease | 66,020 (4.7) | 7,840 (5.7) | **<0.001** |
| Peptic ulcer disease | 27,175 (1.9) | 3,175 (2.3) | **<0.001** |
| Mild liver disease | 77,510 (5.6) | 7,370 (5.4) | 0.14 |
| Diabetes | 145,615 (10.5) | 25,395 (18.5) | **<0.001** |
| Diabetes Complicated | 94,500 (6.8) | 20,910 (15.2) | **<0.001** |
| Hemiplegia/paraplegia | 14,595 (1) | 2,440 (1.8) | **<0.001** |
| Renal disease | 178,585 (12.9) | 33,185 (24.1) | **<0.001** |
| Cancer | 65,620 (4.7) | 7,130 (5.2) | **0.001** |
| Moderate/severe liver disease | 26,780 (1.9) | 1,320 (0.9) | **<0.001** |
| Metastatic cancer | 32,805 (2.4) | 2,770 (2) | **<0.001** |
| AIDS/HIV | 4,110 (0.3) | 235 (0.2) | **<0.001** |
